# Editorial: Expanding the evidence base: the cultural continuum of interventions for eating disorders and related body image difficulties

**DOI:** 10.3389/fpsyt.2026.1980904

**Published:** 2026-10-27

**Authors:** Jennifer Jordan, Gloria Fraser, Eva M. Trujillo-Chi Vacuán

**Affiliations:** 1 Department of Psychological Medicine, University of Otago (Ōtākou Whakaihu Waka), Christchurch, New Zealand; 2 Health New Zealand – Canterbury (Te Whatu Ora- Waitaha), Christchurch, New Zealand; 3 School of Health, Te Herenga Waka – Victoria University of Wellington, Wellington, New Zealand; 4 Research Department, Comenzar de Nuevo A.C., Monterrey, Mexico; 5 Tecnológico de Monterrey Escuela de Medicina y Ciencias de la Salud, Monterrey, Mexico

**Keywords:** body image, cultural continuum, diversity, eating disorders, eurocentric, gender diversity and equity, global evidence, intervention

## Background

The dominance of Eurocentric, White, cisgender, heterosexual perspectives has resulted in limited diversity in research and treatment trials (1, 2). Compared with majority populations, minoritised groups often experience disparities in eating-disorder recognition, access to treatment, and treatment retention (1, 3). Culturally adapted interventions appear to improve outcomes for psychiatric conditions other than eating disorders, compared with non-adapted interventions [see (4)]. Without evidence, the applicability of Eurocentric models for eating disorders for Indigenous and ethnic minoritised cultures across the world cannot be assumed. Recently highlighted iatrogenic treatment harms in eating disorder treatment (5) are relevant here. Culturally responsive adaptations may be important to reduce the risk of perpetuating long-established inequities and potential harms for minoritised groups and cultures outside dominant Eurocentric eating disorders paradigms. Adaptations need to align with cultural values, knowledge, and healing traditions of culturally diverse groups, as well as with lived experience of diverse subcultures within Eurocentric societies.

This special topic invited papers addressing the potential mismatch of Eurocentric assessment, prevention, and treatment models for minoritised groups and across different cultures for those with eating disorders and related body image difficulties. Included papers span diverse geographical, cultural, and minoritised populations across the Middle East (*n* = 4), Australasia (*n* = 3), Latin America (*n* = 2), the USA (*n* = 2), Italy (*n* = 1), and China (*n=1*).

## Applicability of Eurocentric models or measures in different geographical or ethnic contexts

Acle extended the skin tone trauma model (6)to propose a potential pathway linking colourism to eating disorders risk among Black, Indigenous, and other People of Colour (BIPOC).

Mancilla-Díaz et al.’s systematic review on the influence of beauty ideals for Indigenous communities in Latin America found “a complex cultural negotiation” (p1) between traditional and Western body ideals and noted corresponding limitations of current body image assessment tools.

Three papers examined aspects of body image in Middle Eastern samples. Alwulaii et al. examined social beauty norms, body image, and self-esteem among men and women in Saudi Arabia, identifying gendered differences in how appearance norms were experienced. Taha et al. reported a substantial proportion of Jordanian medical students screening positive for elevated eating disorder risk, with higher Eating Attitudes Test (EAT-26) scores among women and associations between body mass index (BMI) and both eating attitudes and body-shape concerns. Given rapid socioeconomic change and greater exposure to Western thin ideals in the Gulf region, Melisse et al. conducted a psychometric evaluation of the Body Uneasiness Test in clinical and community samples across Gulf Cooperation Council countries, providing preliminary support for its use in the region, while identifying areas requiring further validation.

Wang et al. identified some gut microbiota differences between Chinese women with anorexia nervosa and healthy controls, but no robust associations with clinical features, underscoring the value of diverse samples, the need for cautious interpretation and further research.

Eating disorder risk and related psychosocial processes among transgender and gender-diverse people were explored in two papers. Bonato et al. examined relationships among beauty standards, eating concerns, resilience, and dehumanisation, finding context-dependent associations involving several resilience factors. Ker et al. reported elevated eating disorder risk among trans people in a national cross-sectional survey in New Zealand, particularly among younger participants and those who were Māori, neurodivergent, experiencing functional impairment, and/or material hardship.

## Adapted prevention programmes

Ciao et al. described outcomes of Body Justice, a co-designed universal eating disorders prevention programme for early adolescents in the Pacific Northwest United States. The programme, incorporating culturally tailored content and media representation, was well-liked by students with marginalised identities, although preliminary evidence suggested outcomes varied across identity subgroups.

Trujillo-ChiVacuán et al. presented the protocol for the first pilot randomised controlled trial of the culturally adapted Body Image Program in Mexico, designed primarily to assess feasibility and acceptability and inform a definitive trial of this programme.

## Adapted treatments

Clark et al noted the mismatch between holistic Māori views of wellbeing and Western eating disorder models may insufficiently address cultural and spiritual dimensions, “creating a gap in culturally responsive care for Māori” in New Zealand. The authors propose integrating specialist supportive clinical management (7) with Rongoā Māori (traditional healing) to provide “a holistic, culturally grounded approach … addressing the whole person within their cultural context”.

El Khazen et al. described the rationale and implementation of culturally adapted cognitive behaviour therapy- enhanced (CBT-E) within the first specialised eating disorder programme in the United Arab Emirates, established in 2017. Adaptations addressed linguistic, sociocultural, and religious considerations, including Ramadan fasting, modesty norms, and family involvement.

Thomas et al. explored Christian religion and spirituality among people with lived experience of eating disorders in Australia and New Zealand, finding that religion and spirituality could be sources of struggle as well as support across illness and recovery.

## Recommendations

This special topic underscores the need to expand evidence-based eating-disorder research and treatment to realise the health and wellbeing aspirations of underserved groups. Globalisation, colonial influences, and increasing exposure to Western appearance-focussed values may reshape body ideals through interactions with local cultural values [e.g., see (Mancilla-Díaz et al.)]. Increasing recognition and reported prevalences of body dissatisfaction and eating disorder symptoms across diverse regions further challenge the historical framing of eating disorders as predominantly Western conditions, although important regional evidence gaps remain (8, 9).

Most of these papers drew on Eurocentric assessments, preventative interventions or treatments, but with attention to cultural or community context - an established approach to cross-cultural research and care (10, 11). However, adapted interventions should not be conflated with those grounded in diverse worldviews. Cultural adaptation represents one important approach but does not in itself address the need for interventions developed from diverse cultural knowledges and ways of being.

The cultural responsiveness framework in **Table 1** may help characterise and position different approaches to cultural or subgroup assessment and treatment considerations in future research.

**Table 1:** A cultural responsiveness framework^†^ for adapting eating disorders or body image assessment, preventative or treatment strategies.

Extent of adaptation	Definition
Culturally sensitive application of evidence-based models	Eurocentric evidence-based approaches delivered with minimal cultural adaptation, while recognising and responding to the individual’s personal, clinical, and cultural characteristics and delivery needs. Consistent with the three-legged stool model (12).
Adapted evidence-based measures or strategies	Evidence-based tools or strategies are adapted to some extent for the specific culture or subgroup i.e., language is translated and the adaptation may incorporate relevant values and concepts from the culture or subculture (11, 13).
Culturally developed treatments	Culturally developed tools or treatment strategies that emerge from within the (sub) cultural values and worldviews (e.g (14)). The intervention may be informed by some elements of Western-derived evidence-based models. This culturally-devised model would be developed and delivered within services developed for those specific (sub) cultures or groups, typically by members of that community (see (15)).

^†^ A cultural responsiveness continuum has also been used in relation to extent of cultural focus in research methodologies (16).

Continued efforts are needed to amplify diverse voices globally by identifying and addressing the structural barriers that limit research and publication from under-represented regions and subgroups affected by body dissatisfaction and eating disorders.
